# Spontaneous pneumothorax associated with primary lung cancer

**DOI:** 10.1186/1749-8090-10-S1-A242

**Published:** 2015-12-16

**Authors:** Walid S Abu Arab, Ahmed M Abdel-Aziz, Abdel-Maguid M Ramadan

**Affiliations:** 1 Cardiothoracic Surgery Department, Faculty of Medicine, University of Alexandria, Alexandria, Egypt

## Background/Introduction

Spontaneous pneumothorax in association with primary lung cancer is infrequent. No exact mechanism is settled for its occurrence. However, it could be either a primary manifestation or complication for a primary lung cancer.

## Aims/Objectives

The aim of this retrospective study is to search for the association of pneumothorax with primary lung cancer.

## Method

Retrospective revision of the files of the patients who presented with spontaneous pneumothorax and admitted to the Cardiothoracic Surgery Department - Main University Hospital of Alexandria, Egypt during the period from January 2005 to January 2015 was performed. Files were searched for association of spontaneous pneumothorax with primary pulmonary carcinoma. Patients who had spontaneous pneumothorax in association with pulmonary metastases were eliminated.

## Results

Four patients have been found in our medical records. Two of them (50%) had advanced squamous cell lung cancer (stage IIIB) and were referred to Oncology Department to receive treatment following pleural drainage with intercostal tube. One of the three (25%) discovered the pulmonary carcinoma following surgery for recurrent spontaneous pneumothorax through bullectomy. This patient received formal lobectomy. Fourth patient had a bronchoalveolar carcinoma that discovered accidentally intra-operatively during bullectomy.

## Discussion/Conclusion

Pneumothorax as a first manifestation of lung cancer is rare. It could be an ominous sign and usually carries bad prognosis. Recurrent spontaneous pneumothorax in old, heavy smoker patient or patient with emphysema should raise the suspicion of presence of pulmonary carcinoma.

